# The Impact of Gene–Environment Interaction on Alcohol Use Disorders

**DOI:** 10.35946/arcr.v34.3.06

**Published:** 2012

**Authors:** Danielle M. Dick, Kenneth S. Kendler

**Affiliations:** **Danielle M. Dick, Ph.D.,***is an associate professor, in the Department of Psychiatry, Virginia Institute for Psychiatric and Behavioral Genetics, Virginia Commonwealth University, Richmond, Virginia.*; **Kenneth S. Kendler, M.D.,***is a professor, in the Department of Psychiatry, Virginia Institute for Psychiatric and Behavioral Genetics, Virginia Commonwealth University, Richmond, Virginia*.

**Keywords:** Alcohol use disorders (AUDs), genetic factors, environmental factors, genetic and environment interactions, twin studies, statistical models, stress, literature review

## Abstract

This article describes three types of gene–environment interactions and the challenges inherent in interpreting these interactions. It also reports on what is known about gene–environment interactions in the field of alcohol use disorders (AUDs). Twin studies of the interaction of genetic and environmental influences on AUDs have resulted in relatively consistent findings and have suggested general mechanisms for interaction effects. These studies generally find that environments that exert more social control (e.g., higher parental monitoring, less migratory neighborhoods, etc.) tend to reduce genetic influences, whereas other environments allow greater opportunity to express genetic predispositions, such as those characterized by more deviant peers and greater alcohol availability. Conversely, the gene–environment literature that has been developed surrounding specific genes has focused largely on the role of stress as a moderator of genetic effects.

This article explores interactions between genetic and environmental effects on alcohol use disorders (AUDs). Two contrasting ideas define what it means to have genes and environment interact. The first approach—the one that this article will focus on—is a statistical perspective. This approach is based on statistical models in which genetic and environmental factors are sometimes measured indirectly (i.e., latent variable modeling—often in twin studies) and sometimes directly via molecular methods (examples of both kinds of interactions are provided below). The statistical approach does not consider the underlying biological process. Rather, it is based on observing processes from afar and modeling them.

The second approach is based on a biological or molecular perspective. The early work by Jacob and Monod on the operon model of gene regulation established that environmental effects can profoundly influence gene expression (Morange 1998). For example, by switching the source of food for bacteria (e.g., from glucose to lactose), researchers can activate a new set of genes that metabolize the lactose molecule. This is another way of thinking about how genes and environment “interact” but one that differs rather dramatically from the statistical viewpoint. From this perspective, the term interact refers to a biological process, measuring environmental exposures in biologically meaningful ways and looking at processes such as gene expression.

Statistical interactions do not equal biological interactions. In fact, any neurobiological system involves multiple gene products interacting with each other, such as components of signaling cascades, neurotransmitters and their receptors, or degradative enzymes. The world of biology seems like nothing but interactions of one molecule with another. Some biologists take this to mean that when we look at the effect of genetic variation, we should see interactions everywhere and that most gene effects involve such interactions. However, this is not true. A large corpus of work in statistical genetics in tractable organisms consistently has shown that most genetic effects look additive (Mather and Jinks 1982). Further explanation of this is beyond the scope of this article. In general use, the term interact sometimes only means “to act together.” This is consistent with the technical concept of an additive model in which the main effects of genes and environment interact. In this article, the term interact will refer to its technical statistical meaning.

Examining gene–environment interactions from a statistical perspective is exemplified by the work of the statistician Ronald Fisher and best expressed in the development of the analysis of variance. In this highly influential statistical technique, as explained in any standard statistical textbook, Fisher posited an approach that first took into account main effects. For example, by studying the height of a particular plant 10 weeks after planting, one could examine the effect of the two different plant strains (reflecting genes) and the two different fertilizers (reflecting the environment). This would produce a main effect for each variable. Beyond this, one would look for a gene–environment (or more technically a “strain by fertilizer”) interaction. This interaction would reflect any explanatory power left over after accounting for the main effects. In many such cases, as noted above, no significant interaction is detected. That is, research shows the effects of genes on the phenotype and the effects of environment on the phenotype and no significant interaction. This is what statisticians will call an additive model—one in which the effects of genes and environment just add together.

If research does detect a significant gene-by-environment interaction, the effects of genes and environment on the phenotype (e.g., plant height) are not independent of one another. The impact of genes depends on environmental exposure and the impact of the environment depends on the effect of genes. Note that these two statements are conceptually equivalent. Expressed in yet another way, the central concept of genotype-by-environment interaction is that of conditionality. That is, it is not possible to understand how genes are acting without taking the environment into account, and vice versa.

## Types of Gene–Environment Interactions and Challenges With Their Interpretation

This section will review three examples of gene–environment effects, which are illustrated in figure 1. Figure 1 shows five groups differing in level of genetic liability for a particular trait Y (e.g., symptoms of an alcohol use disorder [AUD]), from low to high. The diamonds represent the group with the lowest liability; the asterisks represent the highest-liability group. The x-axis shows the effect of the environment in five increasing categories. Level 1 reflects a very benign environment that conveys no increase at all on trait Y. As the environment becomes more pathogenic—from levels 2 to 5—it has a progressively greater and greater impact on trait Y.

Panel A in the figure depicts an additive model. The lines all are parallel with one another. Increasing from low-to high-risk environments (i.e., from environments 1 to 5), the increase in the level of Y is the same across all five genotypes. Genes and environment act independently of one another.

Panel B in the figure depicts what is known as a “fan-shaped” interaction. Note that the impact of genes is dependent on the environment, and vice versa. The key characteristic of a fan-shaped interaction is that, in benign environments, the difference in the level of the outcome variable (i.e., Y) as a function of the level of genetic liability is quite modest. That is, genes are not doing that much in a protective environment. However, with increasingly severe environmental exposures, the difference between genotypes increases. (In theory, of course, it does not have to be the case that the genetic differences are more pronounced in adverse environments than in benign environments. It could be that under very adverse conditions the environment becomes all important, but under more normative environmental conditions there is opportunity to see genetic differences.) Genes have a much more potent impact on the phenotype in a stressful environment. Another useful way to conceptualize such fan-shaped interactions is to see that genes in this context do two different things. First, they set the mean level of genetic liability. Second, they affect an individual’s sensitivity to the impact of the environment.

Figure 3 depicts a crossover interaction, in which the order of genetic effects changes as a function of the environment. Those at lowest risk in environment 1 are at highest risk in environment 5. One would expect the environment, on average, to have an impact on the phenotype because the average level of risk for individuals in environment 5 (the highest risk environment) will be substantially greater than the average level of risk in the most benign environment (environment 1). However, in general, the main effect on the genotype is limited in this situation, because of a balance between the risk-decreasing effects in benign environments and the risk-increasing effects in malignant environments.

The literature surrounding plant and animal genetics indicates that fan-shaped interactions generally are more common than crossover interactions (Lynch and Walsh 1998; Mather and Jinks 1982). They are more difficult to interpret, however, because a statistical transformation of the scale of measurement can make many fan-shaped interactions disappear. That is, by examining the raw scale scores for a particular trait, it is possible to find significant evidence for a fan-shaped interaction. However, applying statistical analysis (i.e., logarithm or square-root transformation) of the scale scores often causes the interaction to disappear (Lynch and Walsh 1998; Mather and Jinks 1982).

Determining whether the interaction is indeed legitimate is a complicated question. Part of the answer has to do with the degree of “grounding” of the particular scale of measurement that one is examining. In studies of AUD risk, the particular measures are relatively arbitrary and might reflect the number of endorsed *Diagnostic and Statistical Manual, Fourth Edition* (DSM–IV) criteria. In this case, it is difficult to strongly argue that the number of DSM criteria is inherently more real than the square root of those numbers. This adds an extra interpretational difficulty to many analyses of genotype–environment interaction that do not carefully explore the degree to which transformations of the scale of measurement can make the interactions disappear.

A related problem is the common use of logistic regression in the analyses of genotype–environment interaction. Logistic regression is a convenient statistical tool when the dependent measure is dichotomous—such as whether an individual does or does not have a particular disorder. However, logistic regression involves a logarithmic transformation of the probability of being affected. This profoundly changes the nature of relationships between variables, because two variables that multiply as regular numbers will add together when logarithms are applied. The interpretation of interactions that relies solely on logistic regression therefore is rendered relatively treacherous. The interpretation of these results depends in part on a long argument in the epidemiological literature about whether the additive or the multiplicative model of risk is most appropriate.

Eaves (2006) simulated the effect of candidate genes and specific environmental factors in predicting a normally distributed continuous variable using a purely additive model (as in panel A of the figure). The resulting continuous results were dichotomized at a particular threshold value, and the dichotomized data were analyzed by logistic regression. Depending on the nature of the simulation, genotype–environment interaction was detected (spuriously) in 70 to 100 percent of the simulations. These results indicate that genotype–environment studies that detect interactions using logistic regression for dichotomous dependent measures should be interpreted with caution. It is quite challenging in such studies to determine whether the result is valid or an artifact of the statistical measures used. Kendler and Gardner (2010) have further explored this puzzling question of the interpretation of interactions.

## Gene–Environment Interaction in the Field of AUDs

### Examples of Latent Gene–Environment Interaction

Alcohol research is an area where one might imagine gene–environment interaction effects to be particularly important in etiological models because, by definition, exposure to alcohol is a necessary condition for the eventual development of alcohol-related problems. For example, one of the most widely replicated genetic associations with alcohol dependence is the protective role of a genetic variant responsible for the enzyme aldehyde dehydrogenase (i.e., *ALDH2*).^1^ The enzyme produced by a genetic variant in *ALDH2* is comparatively inactive, interfering with the metabolism of alcohol, which leads to facial flushing and other aversive physiological symptoms when alcohol is consumed (Shen et al. 1997). Accordingly, the association between this gene and risk for alcohol dependence necessarily operates through alcohol exposure. Environments that modify the extent of exposure to alcohol therefore would be predicted to moderate the degree to which genetic variability is important. In the extreme, this becomes obvious. If there is no alcohol in the environment, then genetic risk factors for AUDs cannot, by definition, express themselves.

A growing twin literature provides evidence that a variety of different environmental domains that influence access to alcohol and opportunity to engage in alcohol use moderate the importance of genetic influences. One of the earliest illustrations of gene–environment interaction in the area of substance use research demonstrated that genetic influences on alcohol use were greater among unmarried women, whereas having a marriage-like relationship reduced the impact of genetic influences on drinking (Heath et al. 1989). Religiosity also has been shown to moderate genetic influences on alcohol use among female subjects, with genetic factors playing a larger role among individuals without a religious upbringing (Koopmans et al. 1999).

Adolescent alcohol use also seems to be particularly influenced by gene–environment interactions, as might be expected because most adolescents are moving through a developmental period when adult guardians still exert a fair degree of control over their environment. Genetic influences on adolescent substance use are enhanced in environments with lower parental monitoring (Dick et al. 2007*b*), and easy availability of alcohol (Kendler et al. 2010), as well as in the presence of substance-using friends (Dick et al. 2007*a*; Harden et al. 2008; Kendler et al. 2010). Socioregional or neighborhood-level influences also have been shown to moderate the importance of genetic influences on substance use. Genetic influences for late-adolescent alcohol use (and early-adolescent behavior problems, which are genetically correlated) are enhanced in urban environments, communities characterized by greater migration, and neighborhoods with higher percentages of slightly older adolescents/young adults (Dick et al. 2001, 2009*a*; Rose et al. 2001). These community-based moderation effects presumably reflect differences in the availability of alcohol, role models, neighborhood stability, and community-level monitoring across different areas.

It is likely that many of the important moderating effects of the environment associated with alcohol use and related externalizing behavior reflect differences in social control and/or opportunity, resulting in differential expression of individual predispositions (Shanahan and Hofer 2005). Accordingly, the relevant environments are likely to vary across developmental stage. There is some indication of this in the Finnish twin data, where parental monitoring showed significant moderating effects on substance use starting earlier in adolescence (age 14), whereas the moderating role of peer substance use was not apparent until later in adolescence (age 17). More research in this area is necessary to delineate the developmental periods during which specific environments are critical because alcohol use patterns (and their etiological influences) are dynamic across the transition from adolescence to young adulthood. This also is likely to be true across stages of adulthood, although comparatively little research has been dedicated to this area.

## Examples of Gene–Environment Interaction Involving Molecular Variants

As explained above, gene–environment interaction can be detected through the study of genetic influences that are inferred via comparisons of different types of relatives (such as twins) (i.e., latent genetic influences), or through the study of specific measured genes by molecular techniques. Gene–environment interactions modeled latently have the advantage of providing information about the overall genetic effect averaged across the entire genome but tell nothing about the specific underlying biology. Studies of specific genes have the advantage of providing information about the underlying biology, but they are (at this point) largely limited to studying single genes in a system in which there are likely to be hundreds of genes involved.

The literature surrounding specific gene–environment interactions in the area of alcohol use has developed largely independently of the latent gene–environment interaction literature reviewed above. Much of the literature examining measured gene–environment interactions with alcohol use outcomes has focused on stress, which was measured in a variety of ways, a moderator of specific genetic influences. The relationship between stress and alcohol use is complex, with human experimental studies, animal studies, and epidemiological studies all yielding equivocal evidence as to whether stress induces alcohol use (Schwandt et al. 2010; Veenstra et al. 2006). However, the gene–environment interaction literature presupposes that one of the reasons for these disparate findings may be that stress is more likely to induce alcohol use and problems in people who are genetically vulnerable, similar to the literature surrounding the experience of stressful life events and the onset of depression (Kendler et al. 1995).

A number of studies have tested for interactions between alcohol-related outcomes and various measures of stress with the genetic variation for length of the promoter region of the serotonin transporter gene (*5-HTTLPR*) (i.e., whether the genetic variant [allele] for long or short promoter region is associated with stress and alcohol use). Two studies found enhanced risk associated with the short allele in the presence of a stressful environment. Covault and colleagues (2007) found that the short allele was associated with more frequent drinking and heavy drinking as well as drug use in college students if they had experienced multiple negative life events in the past year. Kaufman and colleagues (2006) found that the short allele conferred vulnerability to early alcohol use, and that this effect was stronger among maltreated children. Conversely, in the Mannheim Study of Children at Risk, the long allele was associated with more hazardous drinking in males among those exposed to high psychosocial adversity, as defined by early psychosocial stress and/or current life events (Laucht et al. 2009). In a study of Swedish adolescents, having two different alleles (i.e., being heterozygous) at the long/short polymorphism was associated with a higher intoxication frequency in the presence of neutral or bad family relations, which is biologically unlikely (Nilsson et al. 2005). Accordingly, the genetic model associated with the interaction has been inconsistent across studies, and the primary outcomes and measures of the experience of stress have varied considerably.

A more consistent picture has emerged from studies using experimental manipulations of the environment. In a unique prevention study testing for gene–environment interaction associated with the serotonin transporter gene, Brody and colleagues (2009*b*) found that youth carrying the short allele were more likely to initiate high-risk behavior (including alcohol and marijuana use, as well as sexual behavior) over time if they were in the control condition rather than the prevention condition. Similarly, short allele carriers showed increases in substance use over time, but this association was reduced when youth received high levels of involved-supportive parenting (Brody et al. 2009*a*, *b*). Related studies in monkeys indicate that the short allele is associated with higher baseline alcohol consumption (Barr et al. 2004) and increased aggression (Suomi 2006) under conditions of peer rearing (a stressful environment) compared with mother rearing. These studies suggest that experimental manipulation of the environment may be more likely to yield replicable interaction effects than observational designs, as previously has been argued from a statistical perspective (McClelland and Judd 1993). Interaction effects associated with experimental manipulations of the environment also may be more robust because interventions often operate across a variety of environmental domains (e.g., by influencing parenting processes, peer interactions, and equipping individuals with personal tools that are applicable across a variety of settings). Thus, any interaction effects that are detected may be more likely to be replicated for reasons similar to why twin studies, which examine aggregate genetic effects, are more likely to be replicated (discussed further below).

A few studies have evaluated gene–environment interactions with a variant of the gene for the dopamine type 2 receptor (i.e., the *DRD2* Taq1A polymorphism, which actually is located in the neighboring gene *ANKK1*). These studies have suggested that DRD2 A1 carriers show higher alcohol-related problems in the presence of stress (Bau et al. 2000; Madrid et al. 2001) and have higher novelty seeking when their child-rearing environment was assessed as punitive (Keltikangas-Jarvinen et al. 2009). Similarly, there is a small literature surrounding a genetic variant for the enzyme monoamine oxidase (MAO) (i.e., the MAOA polymorphism), adversity, and alcohol-related outcomes. MAO degrades serotonin, dopamine, and norepinephrine, which are all involved in the stress response. One study found a main effect of the MAOA promoter polymorphism on the risk for substance use disorders and an interaction with parenting (Vanyukov et al. 2007). In another study, the MAOA low-activity allele was associated with alcoholism, and particularly with antisocial alcoholism, but only among women experiencing childhood sexual abuse (Ducci et al. 2008). In yet another small study of female adolescents, the long variant increased risk for alcohol-related problems in the presence of an unfavorable environment (as defined by poor family relations or maltreatment/abuse). However, this effect was opposite that reported in the other studies (Nilsson et al. 2008). Accordingly, the association between this genotype and alcohol-related outcomes remains equivocal.

A few notable efforts have been made to extend the measured genotype–environment interaction literature in the field of alcohol-related outcomes in new directions. One such effort tested for moderation effects associated with brain gene expression in rodent models. Evidence in alcohol-preferring rats suggested that variation in the corticotrophin-releasing hormone releasing receptor 1 (*crhr1*) gene was associated with increased sensitivity to relapse into alcohol seeking induced by environmental stress (Bjork et al. 2010). The Mannheim Study of Children at Risk found an association between variants in *crhr1* and higher rates of heavy drinking and more drinking per occasion among 15-year-olds if they had experienced a greater number of negative life events over the previous 3 years (Blomeyer et al. 2008). An extension of this study followed up the adolescents at age 19 and also found that this gene interacted with stressful life events to predict both drinking initiation in adolescence and progression to heavy alcohol use in young adulthood (Schmid et al. 2010).

In addition, Dick and colleagues have attempted to bridge the gap between the latent gene–environment interaction literature and specific measured gene–environment interactions by developing hypotheses about the risk associated with genes. On the basis of twin studies suggesting that genetic influences on adolescent substance use are moderated by parental monitoring (Dick et al. 2007*b*) and peer substance use (Dick et al. 2007*a*), the researchers tested for moderation of the association of two genes associated with adult alcohol dependence in the Collaborative Studies on Genetics of Alcoholism project. The two genes were for the γ-aminobutyric acid receptor (GABAR) subunit α-2 (*GABRA2*) (Edenberg et al. 2004) and the cholinergic muscarinic 2 receptor (*CHRM2*) (Wang et al. 2004). The researchers found evidence for gene-by-interaction effects in the direction predicted by the twin studies, namely genetic effects were enhanced under conditions of lower parental monitoring (Dick et al. 2009*b*) and higher peer-group antisocial behavior (Latendresse et al. 2011).

## Conclusions

Although there is a burgeoning literature surrounding gene–environment interactions in the field of alcohol use and related disorders, far more remains to be understood. In general, the findings from gene-by-environment twin studies have been relatively consistent and have suggested general mechanisms for interaction effects. The common theme that emerges across findings of gene–environment interactions from the twin literature is that environments that exert more social control (e.g., higher parental monitoring, less migratory neighborhoods, etc.) tend to reduce genetic influences, whereas other environments allow greater opportunity to express genetic predispositions, such as those characterized by more deviant peers and greater alcohol availability. Conversely, the gene–environment literature that has been developed surrounding specific genes has focused largely on the role of stress as a moderator of genetic effects. Clearly, there is a disconnect between these literatures. In addition, it is likely that there are other important mechanisms of gene–environment interaction effects in relation to alcohol use and the development of problems. Many other variables, both individual and psychosocial, are known to affect drinking behavior, such as beliefs about alcohol, self-esteem, school attitudes, parental expectancies and messages surrounding alcohol use, and family disruption (Donovan and Molina 2011). It will be important to integrate these literatures, and the broader basis of etiological findings and associated environmental factors, into theoretical models of how gene–environment interaction effects operate with respect to alcohol use.

Another important area for future research is an expansion of the molecular studies of gene–environment interaction beyond a small number of polymorphisms from a handful of genes that are widely studied in the psychological literature (i.e., *5-HTT, MAOA,* and *DRD2*). The existent studies have been based on small samples, and results have been inconsistent. Although a focus on single genes may help advance theoretical models about particular biological pathways of risk, they face the same challenge (and currently have been met with the same fate) as studies of main effects of individual genes. That is, they have been notoriously difficult to replicate consistently. This is in contrast to the generally robust gene–environment interaction effects that have emerged from studies of latent genetic influences and, previous to that, the robustness of heritability estimates. This likely reflects the difference between studying overall genetic effects, versus specific genes in a complex polygenic system. The field of genetics has moved toward creating polygene scores that aggregate across many genes and show predictive power in cases where individual genes cannot be detected (Purcell et al. 2009). Moving studies of measured gene–environment interaction in this direction, to encompass aggregate genetic risk, may be one way to improve replicability of effects and to enhance cross-fertilization between quantitative and molecular genetic research.

This approach has the potential to advance our understanding of gene–environment effects. Similar to the way that evidence for heritability from twin studies for a given outcome was originally used to justify searching for specific genes involved in that outcome, evidence for gene–environment interactions from twin studies also can be used to develop hypotheses to test for gene–environment interactions associated with specific, identified genes. Change in the overall heritability across environmental contexts does not necessarily dictate that any one specific susceptibility gene will operate in a parallel manner. However, a change in heritability suggests that at least a good portion of the involved genes (assuming many genes of approximately equal and small effect) must be operating in that manner for a difference in heritability by environment to be detected. In this sense, one is “loading the dice” when testing for specific candidate gene-by-environment interaction effects with an environment that already has been shown to moderate the overall importance of genetic influences on that outcome. As additional research begins to clarify how specific genetic variants contribute to risk for AUDs, greater cross-talk between the twin literature, gene-identification studies, and studies testing for measured genotype-by-environment interactions will be critical to producing a more systematic research program aimed at understanding gene-by-environment effects for this critical and socially important condition.

## Figures and Tables

**Figure:**
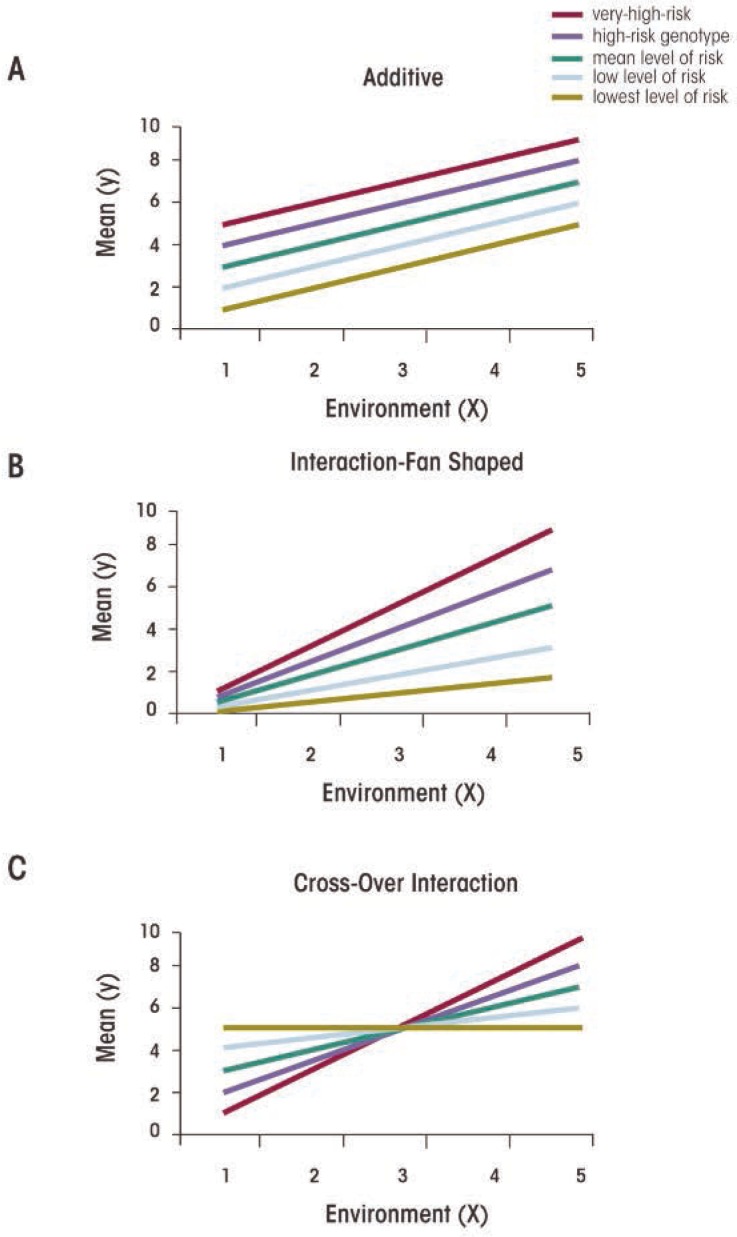
**A–C)** The effect of genes and environment are used to predict the mean level of a quantitative trait Y. The lines depict five different genotypes with varying levels of liability to trait Y (e.g., symptoms of alcohol dependence). The environmental level of risk is depicted on the X-axis and ranges from level 1 (very low risk) to 5 (very high risk). **A)** An additive model of genetic and environmental effects. The key feature of this model is that the lines are all parallel—that is, the increase in the level of trait Y associated with a more adverse environment is the same for all genotypes. **B)** A fan-shaped interaction of genetic and environmental effects on trait Y. **C)** A cross-over interaction of genetic and environmental effects on trait Y.

## Footnotes

1 By convention, gene names in animals are written in upper-case and lowercase and italicized. Gene names in humans are written in all caps and are italicized, whereas the acronyms for the encoded proteins are all caps but not italicized.
